# Humid Heat Equally Impairs Maximal Exercise Performance in Elite Para-Athletes and Able-Bodied Athletes

**DOI:** 10.1249/MSS.0000000000003222

**Published:** 2023-05-22

**Authors:** PUCK ALKEMADE, JOHANNUS Q. DE KORTE, COEN C. W. G. BONGERS, HEIN A. M. DAANEN, MARIA T. E. HOPMAN, THOMAS W. J. JANSSEN, THIJS M. H. EIJSVOGELS

**Affiliations:** 1 Faculty of Behavioral and Movement Sciences, Vrije Universiteit Amsterdam, Amsterdam Movement Sciences, Amsterdam, THE NETHERLANDS; 2 Radboud Institute for Health Sciences, Department of Physiology, Radboud University Medical Center, Nijmegen, THE NETHERLANDS; 3 Amsterdam Institute of Sport Science, Amsterdam, THE NETHERLANDS

**Keywords:** PARALYMPIC, OLYMPIC, TOKYO 2020, HYPERTHERMIA, HEAT STRESS, ADAPTED SPORTS

## Abstract

**Purpose:**

This study aimed to compare the impact of hot-humid environmental conditions on performance outcomes, thermoregulatory responses, and thermal perception during exercise between elite para- and able-bodied (AB) athletes.

**Methods:**

Twenty elite para-athletes (para-cycling and wheelchair tennis) and 20 elite AB athletes (road cycling, mountain biking, beach volleyball) performed an incremental exercise test in a temperate environment (mean ± SD, 15.2°C ± 1.2°C; relative humidity, 54% ± 7%) and a hot-humid environment (31.9°C ± 1.6°C, 72% ± 5%). Exercise tests started with a 20-min warm-up at 70% of maximal heart rate, after which power output increased by 5% every 3 min until volitional exhaustion.

**Results:**

Time to exhaustion was shorter in hot-humid versus temperate conditions, with equal performance loss for para- and AB athletes (median (interquartile range), 26% (20%–31%) vs 27% (19%–32%); *P* = 0.80). AB athletes demonstrated larger exercise-induced increases in gastrointestinal temperature (T_gi_) in hot-humid versus temperate conditions (2.2 ± 0.7 vs 1.7 ± 0.5, *P* < 0.001), whereas T_gi_ responses in para-athletes were similar between conditions (1.3 ± 0.6 vs 1.3 ± 0.4, *P* = 0.74). Para- and AB athletes showed similar elevations in peak skin temperature (*P* = 0.94), heart rate (*P* = 0.67), and thermal sensation score (*P* = 0.64) in hot-humid versus temperate conditions.

**Conclusions:**

Elite para-athletes and AB athletes demonstrated similar performance decrements during exercise in hot-humid versus temperate conditions, whereas T_gi_ elevations were markedly lower in para-athletes. We observed large interindividual variation within both groups, suggesting that in both para- and AB athletes, personalized heat mitigation plans should be developed based on individual thermal testing.

Sports events for elite athletes are increasingly being held in hot and/or humid summer conditions, and exercise under extreme temperatures is expected to be more common in the future because of climate change (1). During exercise in hot-humid conditions, heat dissipation through convection, radiation, and evaporation is restricted and may be insufficient to compensate the body’s metabolic heat production. The resultant heat strain induces cardiovascular challenges, as well as changes in central nervous system function and muscle metabolism, which may all contribute to a substantial reduction in aerobic exercise performance (2,3).

Performance decrements in the heat have been investigated extensively in able-bodied (AB) athletes (4–7). However, to our knowledge, heat-related performance losses have not been quantified in para-athletes. Many para-athletes may demonstrate amplified performance losses compared with AB athletes, because impairments such as a spinal cord injury, limb deficiency, or cerebral palsy can affect thermoregulatory abilities (8–10). On the other hand, many para-athletes perform either upper-body exercise or lower-body exercise at a lower intensity and may therefore produce less metabolic heat than AB athletes, potentially reducing overall heat strain and the associated performance loss. Notwithstanding, the para-athlete population is highly heterogeneous, which may cause large interindividual variability in the response to exercise under heat stress.

The aim of this study was to compare the impact of hot-humid environmental conditions on performance outcomes, thermoregulatory responses, and thermal perception during exercise between elite para- and AB athletes. We hypothesized that para-athletes may demonstrate less heat-related performance loss than AB athletes, as exercise may be associated with a lower heat production and therefore lower heat strain in para- versus AB athletes.

## METHODS

### Participants

In this study, we included 20 elite para-athletes and 20 elite AB athletes. Eleven para-athletes and 12 AB athletes were endurance trained, whereas 9 para-athletes and 8 AB athletes were mixed trained (11) (Table 1). All athletes were Dutch, recruited via TeamNL (the Olympic division of all Dutch sports federations) infrastructures, and competing at an international level. None of the participating athletes were heat acclimatized. Twelve of the 21 female participants used contraception, whereas 4 did not, and 5 did not report. The study was carried out in accordance with the Declaration of Helsinki and was approved by the Medical Ethical Committee of the Radboud University Medical Centre (no. 2018-4640). All participants gave their written informed consent before the testing procedures. The data that support the findings of this study are available from the corresponding author upon reasonable request.

**TABLE 1:** Characteristics of PARA and AB athletes.

	PARA (*n* = 20)	AB (*n* = 20)	*P*
Age (yr)	29 ± 10 (16–50)	27 ± 4 (21–36)	0.34
Sex (male/female)	11/9 (55% male)	8/12 (40% male)	—
Height (cm)	175 ± 11 (160–194)	181 ± 14 (162–211)	0.18
Body mass (kg)	67 ± 11 (47–87)	71 ± 15 (52–106)	0.30
Sports disciplines	Para-cycling, *n* = 11 Wheelchair tennis, *n* = 9	Road cycling, *n* = 7 Mountain biking, *n* = 5 Beach volleyball; *n* = 8	—
Impairment types	Lower limb deficiency, *n* = 9 (unilateral *n* = 5, bilateral *n* = 4) Spinal cord injury (paraplegia), *n* = 4 Visual impairment, *n* = 2 Other physical impairments*^a^*, *n* = 5	—	—

Data are presented as mean ± SD (range).

*^a^*Peripheral neuropathy (*n* = 2), connective tissue disorder (*n* = 1), limb malformation (*n* = 1), spastic hypertonia (*n* = 1).

PARA. para-athletes.

### Study design

The current study is part of the ThermoTokyo research project, of which the rationale and design have been described previously (7,12). Participants performed two personalized incremental exercise tests in a climate chamber. All AB athletes and 4 para-athletes performed lower-body exercise, whereas 16 para-athletes performed upper-body exercise. The first exercise test was conducted in temperate conditions (ambient temperature, 15.2°C ± 1.2°C; relative humidity, 54% ± 7%; wet-bulb globe temperature, 11.9°C ± 0.8°C), which was then repeated in the second test under hot-humid conditions, as expected during the Tokyo 2020 Olympic and Paralympic Games (ambient temperature, 31.9°C ± 1.6°C; relative humidity, 72% ± 5%; wet-bulb globe temperature, 28.8°C ± 0.9°C). Visits took place at the same time of day, separated by 7 (6–7) d (median (interquartile range)). Participants were instructed to, preceding both tests, refrain from strenuous exercise (24 h) and consumption of alcohol or caffeine (12 h), replicate their diet from awakening onwards, consume their last meal ≥3 h before, and consume ~500 mL of water in the 2 h before. During both tests, they wore the same sports clothing and were not allowed to drink. All tests were performed outside of the summer months (October–April).

### Personalized incremental exercise test

Exercise tests were performed on a cycling ergometer (Lode ergometer, Lode B.V., Groningen, the Netherlands (lower-body exercise) or TechnoGym Top Excite+700i, TechnoGym, Cesena, Italy (upper body exercise)) or on the participant’s personal (hand) bike installed in a stationary device (Tacx Neo Smart T2800 or Tacx Booster T2500, Tacx B.V., Wassenaar, the Netherlands) or Cyclus 2 (RBM elektronik-automation, Leipzig, Germany). After the participants entered the climate chamber, cycling ergometer settings were adjusted to fit the participant. Then, participants rested for 5 min in seated position (i.e., baseline), after which the 20-min warm-up phase started. All AB athletes started the warm-up at 100 W, whereas para-athletes started at variable power outputs (30–100 W) based on the estimated individual exercise capacity. After 3 min, power output was gradually adjusted to reach 70% of the participant’s maximal heart rate, obtained from training data or a previous graded exercise test. Further power output adjustments were made each minute until a stable target heart rate was reached. Power output was then kept constant for the remaining minutes of the warm-up phase. After the 20-min warm-up phase, the incremental phase started, in which power output was increased every 3 min by 5% of the power output corresponding to 70% of the maximal heart rate. Exercise was continued until volitional exhaustion. This personalized exercise protocol (i.e., the workloads during the warm-up and incremental stage) was determined in the temperate condition and repeated in the hot-humid condition. Participants were instructed to maintain a cadence of 80–100 rpm throughout the protocol. Ambient temperature, relative humidity, and wet-bulb globe temperature were measured using a portable climate-monitoring device (Davis Instruments Inc., Hayward, CA).

### Measurements

Gastrointestinal temperature (T_gi_) was continuously measured using a validated telemetric temperature capsule system (myTemp, Nijmegen, the Netherlands) (13,14). Participants ingested the capsule 3.2 (3–3.5) h before both study visits. Skin temperature of the posterior side of the neck (T_sk,neck_) was continuously measured using a wireless temperature sensor (iButton DS1922L; Dallas Semiconductor Corp, Dallas, TX), attached to the skin using Tegaderm Film (Tegaderm, Neuss, Germany). Heart rate was continuously measured using a Polar system (Polar V800; Polar Electro Oy, Kempele, Finland). Body mass (in shorts and underwear, towel-dried) was measured to the nearest 100 g using an electronic weighing scale (Seca robusta 813 scale, Hamburg, Germany) before and directly after the exercise protocol to estimate whole-body sweat rate (WBSR). Perceptual scores were obtained at baseline, every 5 min during the warm-up phase, every 3 min during the incremental phase, and at exercise cessation. Thermal sensation, thermal comfort, and rating of perceived exertion were rated on a 7-point, 4-point, and 15-point scale, respectively (15,16). Thermal sensation ranged from −3 (cold) to +3 (hot), thermal comfort ranged from 1 (comfortable) to 4 (very uncomfortable), and rating of perceived exertion ranged from 6 (very, very light) to 20 (maximal exertion).

### Calculations

Time to exhaustion (TTE) was measured from the start of the warm-up until volitional exhaustion. Peak power output (PPO) was calculated as follows: PPO (W) = workload in last complete step (W) + ((time in last incomplete step (min) ÷ step duration (min)) × step size (W)).

Relative TTE and PPO performance losses in hot-humid relative to temperate conditions were calculated as follows: performance loss (%) = (value hot-humid − value temperate) ÷ value temperate ×100. We also calculated the absolute changes in performance, physiological, and perceptual outcomes in hot-humid relative to temperate conditions (ΔHOT–TEMP).

Total work done was estimated by the following: Total work (kJ·kg^−1^) = (mean power output (W) × exercise time (s) ÷ 1000) ÷ body mass (kg).

Physiological data were averaged over 1 min, from which baseline resting values (last minute before start exercise), and peak values (highest minute) were taken. The difference between T_gi_ and T_sk,neck_ during exercise was calculated as a measure of the core-to-skin temperature gradient.

### Statistical analysis

All data were formatted using MATLAB (R2012b; The MathWorks Inc., Natick, MA). Further (statistical) analyses were performed using R software (version 4.1.1; R Foundation for Statistical Computing, Vienna, Austria) in the Rstudio environment (version 2021.09.0 + 351; Rstudio, Inc., Boston, MA). The level of statistical significance was set at *P* < 0.050. Data were reported as mean ± SD (in case of normal distribution) or median (interquartile range; in case of nonnormal distribution, categorical variables, or <10 data points).

In this study, we used a mixed design, with two between-participant levels (para-athletes, AB athletes) and 2 within-participant levels (hot-humid conditions, temperate conditions). To investigate changes in performance, and physiological and perceptual variables on the within-participant level, we performed a paired samples *t*-test or a nonparametric Wilcoxon signed rank test. To investigate changes in these variables on the between-participant level, we performed a Welch’s *t*-test or a nonparametric Wilcoxon rank sum test. Normality of the data was tested per variable, for each cell of the design. When one or more cells of the design did not follow a normal distribution (Shapiro–Wilk test, *P* < 0.05), Wilcoxon tests were used for all pairwise comparisons of that variable, and data were reported as median (interquartile range). Wilcoxon tests were also used to analyze all categorical variables.

## RESULTS

### Personalized incremental exercise test

Power output during the warm-up phase was 65 (55–85) W for para-athletes and 145 (121–165) W for AB athletes (*P* < 0.001). During the incremental phase, power output was increased every 3 min by 5 (3.2–5) W for para-athletes and 9 (6–10) W for AB athletes (*P* < 0.001). Total work was lower for para-athletes compared with AB athletes in both temperate and hot-humid conditions (Table 2).

**TABLE 2:** Exercise performance and physiological responses in PARA and AB athletes in HOT and TEMP conditions, including within- and between-group pairwise comparisons.

	PARA	AB	PARA vs AB
	Mean ± SD or Median (IQR)	Mean ± SD or Median (IQR)	*P*
TTE (min)	*n* = 20	*n* = 20	
TEMP	54 (41 to 62)	58 (45 to 71)	0.29
HOT	40 (30 to 47)	40 (35 to 51)	0.29
ΔHOT–TEMP	−14 (−19 to −8)***	−13 (−24 to −11)***	0.49
PPO (W)	*n* = 20	*n* = 20	
TEMP	131 (102 to 175)	261 (228 to 321)	**<0.001**
HOT	102 (86 to 143)	225 (182 to 264)	**<0.001**
ΔHOT–TEMP	−20 (−35 to −15)***	−39 (−53 to −29)***	**0.007**
Total work (kJ·kg^−1^)	*n* = 20	*n* = 20	
TEMP	5 (3.4 to 6.4)	10.2 (6.4 to 12.7)	**<0.001**
HOT	2.9 (2 to 4.8)	7.2 (5 to 7.6)	**<0.001**
ΔHOT–TEMP	−1.6 (−2.3 to −0.9)***	−2.6 (−5.6 to −2.1)***	**0.003**
T_gi_, baseline (°C)	*n* = 15	*n* = 19	
TEMP	37.2 ± 0.5	36.9 ± 0.4	0.08
HOT	37.2 ± 0.3	36.9 ± 0.4	**0.043**
ΔHOT–TEMP	0 ± 0.5	0.1 ± 0.3	0.79
T_gi_, rise (°C)	*n* = 15	*n* = 19	
TEMP	1.3 ± 0.4	1.7 ± 0.5	**0.01**
HOT	1.3 ± 0.6	2.2 ± 0.7	**<0.001**
ΔHOT–TEMP	0 ± 0.5	0.5 ± 0.5***	**0.007**
T_gi_, peak (°C)	*n* = 19	*n* = 19	
TEMP	38.5 (38.2 to 38.8)	38.6 (38.2 to 38.9)	0.43
HOT	38.5 (38.1 to 39)	39.3 (38.8 to 39.4)	**0.004**
ΔHOT–TEMP	0.2 (−0.3 to 0.4)	0.6 (0.2 to 0.9)***	**0.006**
T_sk,neck_, baseline (°C)	*n* = 18	*n* = 19	
TEMP	31.9 (31 to 32.7)	31.9 (31.2 to 32.4)	0.82
HOT	34.8 (34.5 to 35.2)	34.8 (34.4 to 35.2)	1.00
ΔHOT–TEMP	3 (2.1 to 3.8)***	3.1 (2.2 to 3.8)***	1.00
T_sk,neck_, peak (°C)	*n* = 18	*n* = 19	
TEMP	33.2 (32.2 to 33.8)	34.2 (33.1 to 34.7)	0.07
HOT	36.6 (36.2 to 36.9)	37.8 (37.1 to 38.1)	**0.006**
ΔHOT–TEMP	3.6 (2.9 to 4.4)***	3.7 (2.6 to 4.6)***	0.94
T_gi_-T_sk,neck_ gradient, mean (°C)	*n = 14*	*n = 18*	
TEMP	5.5 (5.1 to 7.8)	5.4 (4.6 to 5.8)	0.14
HOT	1.6 (1.5 to 2.5)	1.4 (1 to 1.8)	0.07
ΔHOT–TEMP	−4.1 (−5.8 to −3.6)**	−3.6 (−4.8 to −3.1)***	0.17
HR, baseline (bpm)	*n* = 19	*n* = 20	
TEMP	75 (67 to 86)	65 (61 to 80)	0.08
HOT	87 (75 to 98)	71 (67 to 81)	**0.007**
ΔHOT–TEMP	8 (6 to 16)***	10 (1 to 16)	0.64
HR, mean (bpm)	*n* = 20	*n* = 20	
TEMP	151 ± 12	143 ± 11	**0.046**
HOT	157 ± 13	149 ± 10	**0.03**
ΔHOT–TEMP	6 ± 7**	5 ± 8**	0.70
HR, peak (bpm)	*n* = 20	*n* = 20	
TEMP	181 ± 11	175 ± 11	0.13
HOT	184 ± 13	180 ± 11	0.28
ΔHOT–TEMP	4 ± 8 (*P* = 0.057)	5 ± 9*	0.67
WBSR (L·h^−1^)	*n* = 18	*n* = 20	
TEMP	0.6 ± 0.3	0.9 ± 0.3	**0.002**
HOT	0.9 ± 0.5	1.5 ± 0.6	**0.003**
ΔHOT–TEMP	0.3 ± 0.4**	0.6 ± 0.3***	**0.049**

For the within-participant comparisons between hot-humid and temperate conditions, asterisks denote *P* values <0.04. Exact *P* values are given when *P* = 0.06–0.04. *P* values <0.05 are displayed in bold.

*P = 0.04–0.01.

**P = 0.01–0.001.

***P < 0.001.

HOT, HOT-humid; HR, heart rate TEMP, temperate; PARA, para-athletes.

### Exercise performance

**FIGURE 1:**
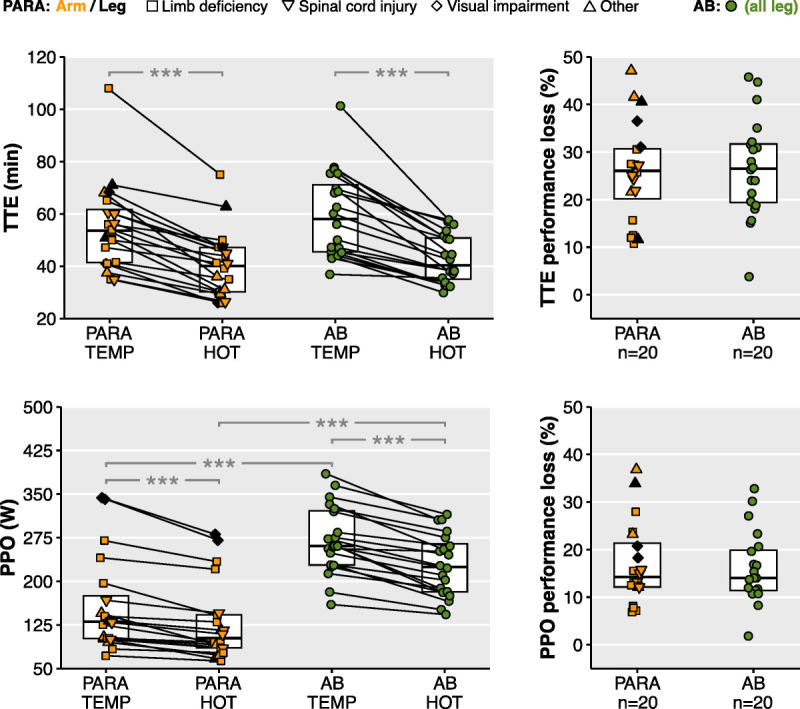
The *left panels* show TTE and PPO in temperate (TEMP) and hot-humid (HOT) conditions, for both para-athletes (PARA) and AB athletes. The *right panels* show the TTE and PPO performance loss in hot-humid vs temperate conditions. For para-athletes, fill and shape indicate exercise mode and impairment type, respectively. Average data are presented as median (IQR). *Asterisks* denote *P* values <0.04, with * for 0.04–0.01, ** for 0.01–0.001, and *** for <0.001, whereas exact *P* values are presented when *P* = 0.06–0.04.

**FIGURE 2:**
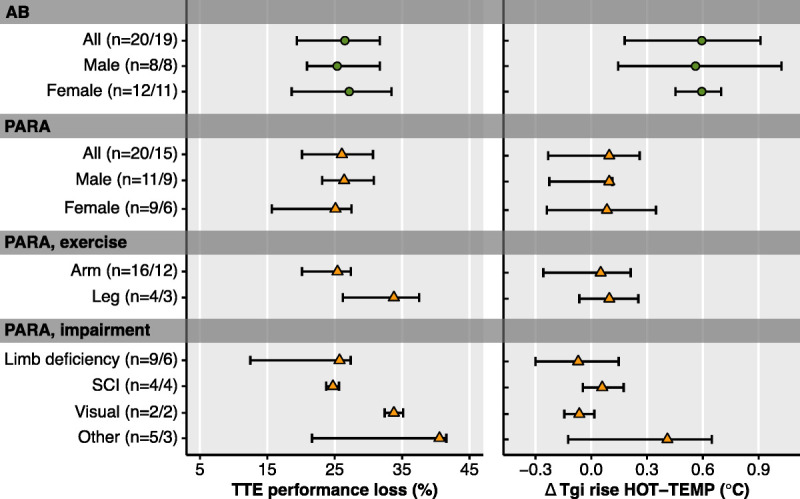
TTE performance loss and the exercise-induced T_gi_ rise in hot-humid (HOT) relative to temperate conditions (TEMP) for subgroups of AB athletes and para-athletes (PARA). Data are presented as median (IQR). Number of datapoints (*n*) are presented as (*n* performance/*n* T_gi_ rise).

TTE did not differ between para- and AB athletes in both conditions, and was shorter in hot-humid versus temperate conditions for both groups (Fig. 1; Table 2). TTE performance loss was 26% (20%–31%) for para-athletes and 27% (19%–32%) for AB athletes, and did not differ between groups (*P* = 0.80). Absolute PPO was lower for para-athletes than for AB athletes in both conditions, and lower in hot-humid versus temperate conditions for both groups (Fig. 1; Table 2). PPO performance loss was 14% (12%–21%) for para-athletes and 14% (11%–20%) for AB athletes (*P* = 0.84). Figure 2 shows performance losses across subgroups of para- and AB athletes.

### Thermophysiological responses

Because of technical issues, T_gi_ data were unavailable for one para-athlete and one AB athlete, and only partial T_gi_ data were available for four para-athletes. T_sk,neck_ data were unavailable for two para-athletes and one AB athlete.

**FIGURE 3:**
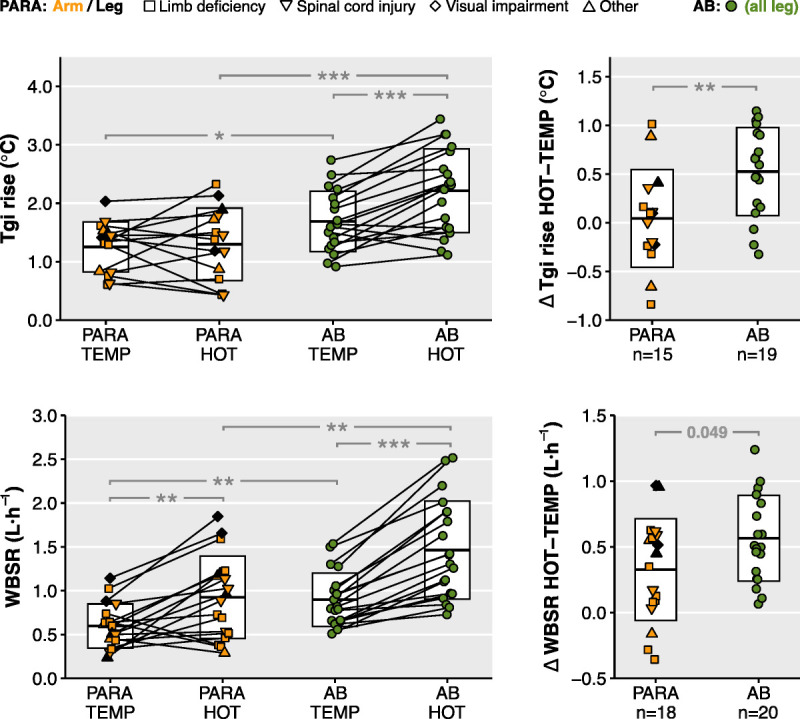
The *left panels* show the exercise-induced T_gi_ rise and WBSR in temperate (TEMP) and hot-humid (HOT) conditions, for both para-athletes (PARA) and AB athletes. The *right panels* show the absolute change in T_gi_ rise and WBSR in hot-humid vs temperate conditions. For para-athletes, fill and shape indicate exercise mode and impairment type, respectively. Average data are presented as mean ± SD. *Asterisks* denote *P* values <0.04, with * for 0.04–0.01, ** for 0.01–0.001, and *** for <0.001, whereas exact *P* values are presented when *P* = 0.06–0.04.

**FIGURE 4:**
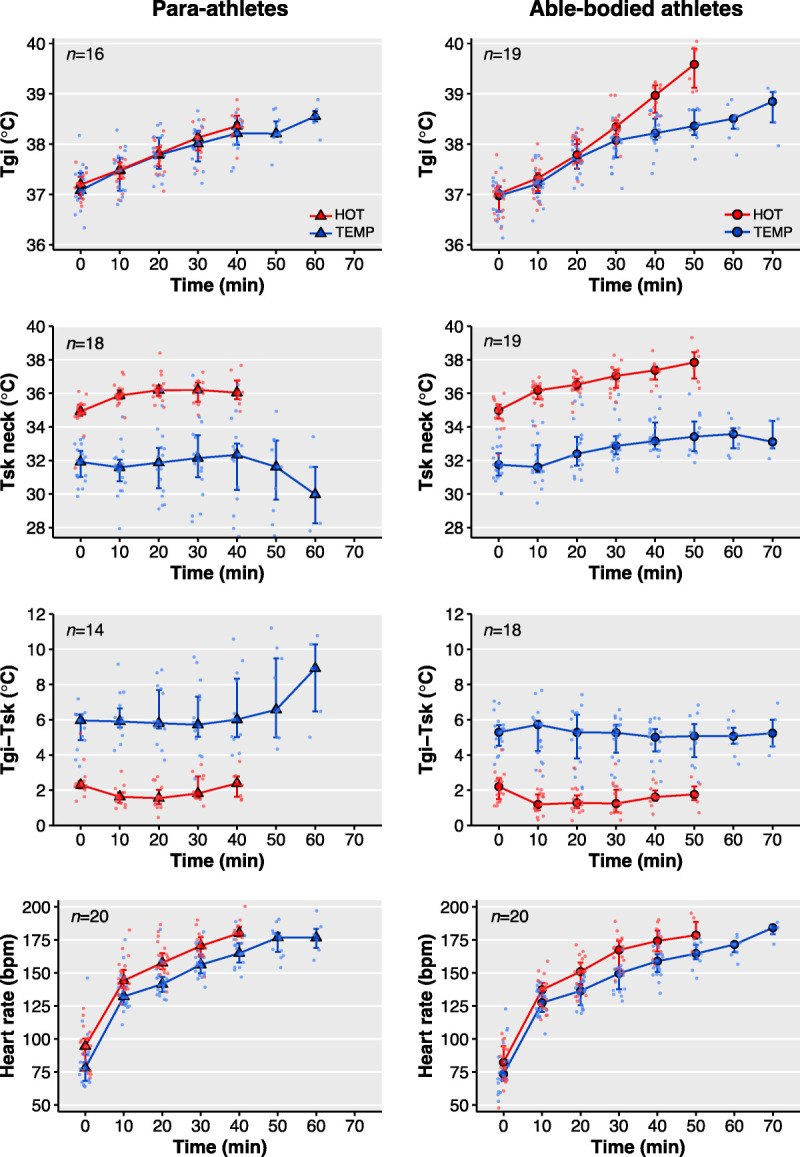
T_gi_, T_sk,neck_, core-to-skin temperature gradient (T_gi_-T_sk_), and heart rate over time in temperate (TEMP) and hot-humid (HOT) conditions, for both para-athletes (*left*) and AB athletes (*right*). Data are presented as median (IQR) for all time points with sample size ≥3.

The exercise-induced rise in T_gi_ was lower for para-athletes than for AB athletes, in both hot-humid (para 1.3°C ± 0.6°C vs AB 2.2°C ± 0.7°C, *P* < 0.001) and temperate (para 1.3°C ± 0.4°C vs AB 1.7°C ± 0.5°C, *P* = 0.01) conditions (Fig. 3; Table 2). The T_gi_ rise for para-athletes was similar in hot-humid and temperate conditions, whereas the T_gi_ rise for AB athletes was greater in hot-humid versus temperate conditions (Fig. 4). Figure 2 shows the impact of hot-humid conditions on the exercise-induced T_gi_ rise across para- and AB subgroups. Peak T_sk,neck_ was lower for para-athletes than for AB athletes in hot-humid (para 36.6°C (36.2°C–36.9°C) vs AB 37.8°C (37.1°C–38.1°C), *P* = 0.006), but not temperate (para 33.2°C (32.2°C–33.8°C) vs AB 34.2°C (33.1°C–34.7°C), *P* = 0.07) conditions. Para- and AB athletes showed similar peak T_sk,neck_ elevations in hot-humid versus temperate conditions (Fig. 4; Table 2). In the para-athletes, average T_sk,neck_ decreased near the end of exercise in temperate conditions (Fig. 4), with the individual responses being highly heterogeneous (see Supplemental Figs. 1 and 2, Supplemental Digital Content, http://links.lww.com/MSS/C873). The athletes showing a clear decrease in T_sk,neck_ during exercise (*n* = 7) had a lower mean power output than the athletes demonstrating a clear increase in T_sk,neck_ (*n* = 8).

Mean exercise heart rate was higher for para-athletes than for AB athletes, in both hot-humid (para 157 ± 13 bpm vs AB 149 ± 10 bpm, *P* = 0.03) and temperate (para 151 ± 12 vs AB 143 ± 11 bpm, *P* = 0.046) conditions. For both groups, mean heart rate was elevated in hot-humid versus temperate conditions (Table 2). Peak heart rate was similar between para- and AB athletes in both hot-humid (para 184 ± 13 bpm vs AB 180 ± 11 bpm, *P* = 0.28) and temperate (para 181 ± 11 bpm vs AB 175 ± 11 bpm, *P* = 0.13) conditions (Fig. 4; Table 2).

WBSR was lower for para-athletes than for AB athletes, in both hot-humid (para 0.9 ± 0.5 L·h^−1^ vs AB 1.5 ± 0.6 L·h^−1^, *P* = 0.003) and temperate (para 0.6 ± 0.3 L·h^−1^ vs AB 0.9 ± 0.3 L·h^−1^, *P* = 0.002) conditions. The WBSR elevation in hot-humid versus temperate conditions was larger in AB athletes than in para-athletes (Fig. 3; Table 2).

### Perceptual responses

**FIGURE 5:**
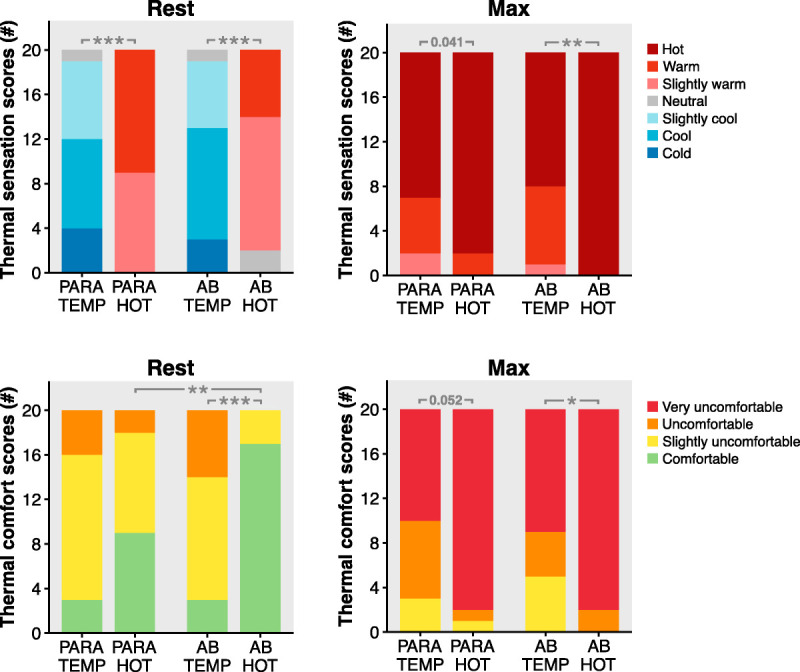
Count (#) of resting and maximal thermal sensation and thermal comfort scores, in temperate (TEMP) and hot-humid (HOT) conditions, for both para-athletes (PARA) and AB athletes. *Asterisks* denote *P* values <0.04, with * for 0.04–0.01, ** for 0.01–0.001, and *** for <0.001, whereas exact *P* values are presented when *P* = 0.06–0.04.

Para- and AB athletes showed similar baseline and maximal thermal sensation scores (Fig. 5; ΔHOT–TEMP para vs AB, *P* = 0.22 (baseline), *P* = 0.64 (max)). In hot-humid conditions, para-athletes felt less thermally comfortable than AB athletes during baseline rest (ΔHOT–TEMP para vs AB, *P* = 0.041), but this difference disappeared during exercise (ΔHOT–TEMP para vs AB, *P* = 0.89). Maximal ratings of perceived exertion were similar among conditions and athlete groups (para: temperate vs hot-humid, 20 (19–20) vs 20 (20–20), *P* = 0.40; AB: temperate vs hot-humid, 20 (19–20) vs 20 (20–20), *P* = 0.67; ΔHOT–TEMP para vs AB: *P* = 0.73).

## DISCUSSION

We compared the impact of hot-humid environmental conditions on performance outcomes, thermoregulatory responses, and thermal perception during exercise between elite para- and AB athletes. Elite para-athletes and AB athletes demonstrated similar heat-induced performance decrements, whereas T_gi_ elevations were markedly lower in para-athletes. Para- and AB athletes showed similar elevations in exercise T_sk_, heart rate, and thermal sensation score in hot-humid versus temperate conditions. Large interindividual variability was observed within both para- and AB athlete groups, with heat-related performance losses ranging from 5% to 50% and exercise-induced T_gi_ rises ranging from −1°C to +1°C in hot-humid versus temperate conditions.

### Exercise performance

Our findings indicate that a hot-humid environment induces considerable performance losses in both para- and AB athletes, resembling previous AB studies (4–7). We are the first to quantify the magnitude of heat-induced performance losses in elite para-athletes. Interestingly, performance was considerably impaired in para-athletes, despite similar T_gi_ in hot-humid and temperate conditions. These findings support the notion that performance impairments in the heat are not only caused by elevations in core temperature but also follow from complex interactions between physiological and perceptual factors (2,3). Indeed, previous AB research also showed that heat-induced performance decrements can occur in the absence of an exacerbated rectal temperature response (4). During incremental exercise to exhaustion in the heat, cardiovascular limitations likely play a major role in the development of fatigue and reduction of maximal aerobic capacity (2,3). We therefore propose that the performance losses in the present study may be attributed to a skin temperature-mediated increase in cardiovascular strain in hot-humid versus temperate conditions (2–4). A high skin temperature narrows the core-to-skin temperature gradient, which increases skin blood flow demands and consequently lowers stroke volume. During submaximal exercise, heart rate increases to maintain cardiac output, but at maximal exercise, cardiac output is likely compromised, accelerating exhaustion (2,3). Indeed, in our study, para- and AB athletes demonstrated similar elevations in neck skin temperature, core-to-skin temperature gradient, and mean heart rate in hot-humid versus temperate conditions. Hence, the skin temperature-mediated increase in cardiovascular strain in hot-humid relative to temperate conditions may have been the primary factor compromising maximal exercise performance in both groups.

Another explanation for the comparable performance losses between para- and AB athletes may relate to the thermal perception during exercise. Previous studies, involving various exercise protocols (self-paced, constant workload, incremental), demonstrated that thermal perception can influence exercise performance independent of core temperature (17–19). During exercise in the heat, skin temperature elevation may aggravate thermal perception and cardiovascular strain, leading to an increased perceived exertion at a given work rate, and thereby a reduction in voluntary exercise capacity (20). In our study, para- and AB athletes demonstrated similar heat-induced elevations in neck skin temperature and heart rate, as well as comparable aggravation of maximal thermal sensation and comfort scores. This was indeed accompanied by a heat-induced increase in perceived exertion for both groups; in hot-humid conditions, maximal perceived exertion scores were similar to that in temperate conditions, even though power output was considerably lower. Thus, the hot-humid environment elicited comparable perceptual responses in para- and AB athletes, which may have impaired exercise performance to a similar extent.

### Heat strain

The hot-humid environment exacerbated the hyperthermic response during exercise in AB athletes but not para-athletes. This may be unexpected because it has been suggested that para-athletes have a reduced thermoregulatory ability due to their impairment, exposing them to a greater risk for hyperthermia and exertional heat illness than AB athletes (9,21,22). However, one should take into account that during training and competition, para-athletes will likely exercise at a lower work rate and with a smaller muscle mass than AB athletes, especially those who are wheelchair-bound, resulting in a lower metabolic heat production. To simulate such a realistic scenario, we implemented a personalized exercise protocol, which allowed all athletes to exercise at a similar relative exercise intensity and to reach their individual maximum. Using this approach, we observed that work rate and thus metabolic heat production were considerably lower in para-athletes compared with AB athletes, presumably explaining their lower peak T_gi_ and WBSR values. The lower WBSR in para-athletes likely resulted from the lower requirement for evaporation, but might also relate to a reduced body surface area for sweating in the para-athletes with an amputation or spinal cord injury (*n* = 13) (23,24). The lower heat strain in para-athletes compared with AB athletes raises doubt whether, in a real-world sport scenario, the risk for excessive hyperthermia and exertional heat illness is actually higher in para-athletes. This question is further justified by our recent study in which we observed that the incidence of exertional heat illness in Paralympic athletes may be lower than expected (25). Nevertheless, it must be recognized that para-athletes may exhibit severe hyperthermia during competition in the heat, especially those performing lower-body exercise at high intensity or those with a high-level spinal cord injury (8–10). Future experimental studies are required to identify which para-athlete subgroups are at highest risk for severe hyperthermia, considering exercise mode, impairment type, and competition demands. Most importantly, we observed large interindividual differences in the heat-induced T_gi_ responses, especially within the para-athlete group, emphasizing the need for individual thermal testing of elite athletes in preparation for competition in the heat.

### Strengths and limitations

Our study was, to our knowledge, the first to investigate heat-related performance losses in para-athletes. This was done within a well-controlled laboratory setting, and in a unique group of elite athletes. Notwithstanding these strengths, some limitations should be considered. First, we included a heterogeneous group of para-athletes. We explored differences in heat-induced performance decrements or T_gi_ responses across para-athlete subgroups, but given the low sample size of these subgroups, we need to interpret this with caution. Athletes with severe thermoregulatory impairments, such as a high-level spinal cord injury, may not be able to adequately dissipate the heat produced during exercise, potentially resulting in severe hyperthermia (8). Future studies should investigate heat-induced performance decrements in this specific para-athlete subgroup. Second, the personalized exercise protocol in the present study differs from real-world sports situations with respect to intensity requirements, behavioral responses (e.g., pacing and tactical decisions), and environmental factors (e.g., solar radiation and wind). This may limit the translation to field settings. However, an observational field study may hinder the direct comparison of performance loss between para- and AB athletes, because of uncontrolled factors such as variations in ambient conditions, or influence of tactical elements. Furthermore, we preferred the personalized incremental exercise test to exhaustion over a time trial approach, to allow comparisons across athletes from various sports disciplines. A time-trial approach may not be appropriate for athletes inexperienced with time trials (e.g., tennis players, beach volleyball players), as there may be a learning effect resulting in pacing adjustments from the first to the second test (26). Altogether, this novel and well-controlled assessment of heat-related performance losses in para-athletes provides a solid basis for future research (in the field).

### Practical implications

This study provides a unique insight into heat-related performance decrements in elite para-athletes and demonstrates how this relates to performance impairments in elite AB athletes. The comparison to AB athletes is valuable, as practical guidelines and recommendations are predominantly based on AB research (27–30). Our findings suggest that para-athletes, like AB athletes, should use heat mitigation strategies to attenuate performance losses in the heat. However, the optimal type of cooling may depend on the thermoregulatory responses accompanying the performance loss. For para-athletes exercising at a low work rate, a high core temperature may rarely be the main factor limiting exercise performance. These athletes might therefore predominantly benefit from cooling techniques that reduce the exercise-induced rise in skin temperature and improve thermal perception (e.g., cooling vest, cold packs), rather than methods that primarily aim to attenuate the rise in core temperature (e.g., ice slurry). However, we observed large interindividual differences within both para- and AB athlete groups, suggesting that individual testing of elite athletes is necessary to develop personalized heat mitigation plans.

## CONCLUSIONS

Hot-humid conditions severely impaired exercise performance in both elite para-athletes and elite AB athletes, as evidenced by reductions in TTE of 26% (20%–31%) and 27% (19%–32%), respectively. AB athletes demonstrated considerable T_gi_ elevations in hot-humid versus temperate conditions, whereas no T_gi_ differences were apparent in para-athletes. Nevertheless, para- and AB groups showed similar elevations in exercise T_sk_, heart rate, and thermal sensation score in hot-humid versus temperate conditions. These findings suggest that both para- and AB athletes should utilize heat mitigation strategies to attenuate performance loss during competition in the heat. Extensive thermal testing in both para- and AB settings is recommended to determine the optimal heat mitigation strategy for each individual athlete.
